# Integrative analysis of salt tolerance in wheat using multi-gradient phenotyping and molecular/physiological validation

**DOI:** 10.3389/fpls.2026.1813443

**Published:** 2026-04-29

**Authors:** Jia-nan Huang, Hong-jin Wang, Yindeng Ding, Guiqiang Fan, Yonghong Gao, Burebiyanmu Wubulikasimu, Zikun Wang, Uzair Ullah, Lubna Khan, Hui Fang, Tianrong Huang

**Affiliations:** 1 Institute of Crop Research, Academy of Agricultural Sciences of Xinjiang Uyghur Autonomous Region, Urumqi, China; 2 School of Materials Science and Engineering, Jingdezhen Ceramic University, Jingdezhen, Jiangxi, China; 3 National Key Laboratory of Crop Improvement for Stress Tolerance and Production, College of Life Sciences, Northwest A&F University, Yangling, Shaanxi, China; 4 College of Biological Science and Engineering, Shaanxi University of Technology, Hanzhong, Shaanxi, China

**Keywords:** hierarchical clustering, phenotypic screening, salt stress, salt tolerance index, TOPSIS, wheat germplasm, ion homeostasis

## Abstract

**Introduction:**

Salt stress is a major constraint on wheat growth and productivity, necessitating efficient methods for identifying salt-tolerant germplasm.

**Methods:**

In this study, 120 wheat varieties were evaluated at the seedling stage under four NaCl concentrations (0, 50, 100, and 150 mM). Key traits, including plant height, root length, germination rate, and salt tolerance index (STI), were measured across two independent replicates. Statistical analyses, including ANOVA, correlation analysis, principal component analysis (PCA), hierarchical clustering, and TOPSIS, were applied to assess phenotypic variation and rank salt tolerance. Representative tolerant and sensitive varieties were further validated through physiological and molecular analyses.

**Results:**

Increasing salinity significantly inhibited growth and germination, with the greatest variation observed at 100–150 mM NaCl. Salt concentration, genotype, and their interaction significantly affected all traits. STI showed strong positive correlations with growth traits. Multivariate analyses revealed stable phenotypic groupings and identified tolerant and sensitive varieties. Physiological and molecular validation demonstrated that tolerant varieties maintained better ion homeostasis, higher antioxidant activity, lower membrane damage, and favorable K⁺/Na⁺ ratios under salt stress.

**Discussion:**

This study establishes an integrated framework combining multi-gradient phenotyping, statistical analysis, comprehensive ranking, and mechanistic validation. The approach provides a reliable and scalable strategy for identifying salt-tolerant wheat germplasm and supports future genetic research and breeding programs.

## 1 Introduction

Soil salinization and alkalinization are major ecological challenges threatening global agriculture (Stavi et al., 2021). Affected by factors such as improper irrigation, changes in groundwater levels, and abnormal climate conditions, the area of salinized arable land continues to expand, resulting in increasingly frequent and severe salt stress risks for crop production systems (Pulido-Bosch et al., 2018a; Eswar et al., 2021; Mohanavelu et al., 2021). Irrigation with poor-quality water accelerates salt buildup in the soil, especially under limited drainage, leading to ion toxicity and osmotic stress for crops (Qadir et al., 2014; Baghel and Tripathi, 2025). Changes in groundwater levels, particularly due to over-extraction or rising water tables, also contribute to secondary salinization in arid and semi-arid regions (Salama et al., 1999; Pulido-Bosch et al., 2018b). In addition, climate anomalies such as increased temperatures and altered precipitation patterns can exacerbate soil salinity by enhancing evapotranspiration and reducing salt leaching (Cai et al., 2015; Corwin, 2021). As a result, salt stress becomes a pervasive constraint on crop development, growth, and productivity in many agricultural systems worldwide (Raza et al., 2019).

Wheat (*Triticum aestivum* L.) is one of the most important food crops worldwide (Shiferaw et al., 2013). Although wheat has a certain degree of tolerance to salt stress, it can still significantly inhibit growth and development and reduce yield potential, particularly during the germination and early seedling stages when plants are more sensitive (EL Sabagh et al., 2021; Zhao et al., 2025). Salt stress not only induces osmotic stress and ion toxicity, but also disrupts nutrient uptake, promotes excessive accumulation of reactive oxygen species (ROS), and exacerbates membrane lipid peroxidation and structural damage, ultimately leading to phenotypic changes such as reduced germination rate, inhibited root elongation, decreased plant height, and lower biomass (Grattan and Grieve, 1998; Zhu, 2001; Gill and Tuteja, 2010; Atta et al., 2023). Therefore, systematically exploring salt-tolerant germplasm and establishing an efficient and reproducible system are crucial for enhancing wheat production in saline-alkali regions and advancing the development of salt-tolerant breeding materials.

Wheat salt tolerance is a typical complex quantitative trait, with its phenotypic expression jointly regulated by genetic background, stress intensity, and their interaction (Liu et al., 2025; Wang et al., 2025b). Traditional screening typically evaluates single traits or uses a single salt concentration, which may overlook stage-specific and dose-dependent responses (Tao et al., 2021). However, this approach can easily overlook the dose-dependent and stage-specific nature of salt tolerance responses (Xu et al., 2024). In fact, wheat’s response to salt stress often exhibits a non-linear pattern, with different phenotypic traits showing varying sensitivity: roots, being directly exposed to the salt environment, are typically inhibited earlier and more severely than aboveground traits, and the germination and seedling growth stage may involve distinct physiological mechanisms (Munns and Tester, 2008; Robin et al., 2016; Negrão et al., 2017). Screening based on a single salt concentration or single trait may fail to distinguish between materials that are “stable under mild stress but rapidly collapse under severe stress” and those that “maintain stable performance across stress levels”, potentially leading to evaluation bias and misjudgment (van Straten et al., 2019; Tao et al., 2021; Masarmi et al., 2023). Therefore, developing a systematic screening process that incorporates multiple salt concentration gradients, evaluates multiple traits jointly, and is supplemented by statistical analysis and comprehensive decision-making methods is essential for improving the efficiency and reliability of salt-tolerant germplasm identification and evaluation.

In recent years, advances in high-throughput phenotyping and data analysis methods have facilitated the application of population-scale computational analysis in germplasm research (Araus and Cairns, 2014; Nguyen and Norton, 2020; Sheikh et al., 2024). Analysis of variance (ANOVA) can quantitatively evaluate the contribution of salt concentration, genotype, and their interactions to phenotypic variation (Omrani et al., 2022); correlation analysis can reveal synergistic relationships as well as trade-oofs among different traits (Tao et al., 2021); principal component analysis (PCA) and clustering help identify major patterns of variation and reveal the population structure (Song et al., 2025); multi-index decision-making methods, such as Top-Intensity Study (TOPSIS), can integrate multiple traits into a single score, enabling quantitative ranking of salt tolerance and identification of superior candidate materials (Taherdoost and Madanchian, 2024). Furthermore, hierarchical clustering trees constructed based on multiple traits can visually illustrate the overall similarity and potential grouping among materials (Arief et al., 2017; Aycan et al., 2024). However, current screening of salt-tolerant wheat germplasm faces two limitations. First, some studies use limited sample sizes or single salt concentration treatments, making it difficult to fully capture the differentiation of salt tolerance responses under varying stress intensities (Tao et al., 2021). Second, the screening process largely remains at the phenotypic level, without verification of the molecular and physiological mechanisms in extreme materials, which limits the applicability of the results for stability assessment, mechanistic interpretation, and subsequent genetic analysis (Rehman et al., 2025). In particular, during salt stress adaptation, the maintenance of root ion homeostasis (e.g., Na^+^ exclusion and K^+^ retention), reactive oxygen species (ROS) scavenging, and osmotic regulation are critical processes (Gill and Tuteja, 2010; Kaur et al., 2024). Therefore, complementing phenotypic screening with verification of root transcription and physiological/ion homeostasis can enhance the mechanistic credibility and interpretability of screening results.

Building on this background, the present study evaluated 120 wheat varieties across four salt concentration gradients (0, 50, 100, and 150 mM), and measured key seedling phenotypic traits, including plant height, root length, germination rate, and salt tolerance index (STI), with two independent experiments, thereby constructing a multi-salt gradient, multi-trait phenotypic dataset. First, descriptive statistics and dose-response analysis were applied to characterize the effects of salt stress on each trait and to compare how differences among varieties changed across varying stress intensities. Next, ANOVA was performed to assess the contribution of salt concentration, genotype, and their interactions to phenotypic variation, while correlation analysis was conducted to examine the relationships among traits. Furthermore, a distance matrix was generated from multi-salt gradient, multi-trait phenotypic data, and hierarchical clustering was performed. PCA and cluster analysis were then combined to reveal the comprehensive phenotypic grouping pattern of the materials. Based on this, the TOPSIS multi-index comprehensive evaluation method was employed to quantitatively rank the salt tolerance of the varieties, enabling the identification of materials with both excellent salt tolerance and extreme salt sensitivity. Finally, to strengthen the mechanistic support for the screening results, this study performed qRT-PCR validation of salt response-related genes in root tissues, alongside measurement of root antioxidant physiological parameters and Na^+^/K^+^ ion homeostasis in representative salt-tolerant and sensitive materials. Overall, this study aims to establish a reproducible and scalable integrated process of “multi-gradient phenotypic assessment of salt tolerance in wheat seedlings, multidimensional statistical analysis, comprehensive ranking and screening, root mechanism verification”, to provide a reliable framework for the efficient identification of salt-tolerant germplasm, as well as for subsequent genetic analysis, and utilization in salt-tolerant breeding programs.

## 2 Materials and methods

### 2.1 Experimental materials

This study used 120 wheat varieties (or lines) as experimental materials. These materials were sourced from the Crop Research Institute of Xinjiang Academy of Agricultural Sciences, and represent diverse genetic backgrounds with potential variation in salt-alkali tolerance. Germination and seedling growth traits were evaluated under control and salt stress conditions to evaluate salt tolerance and identify candidate varieties with superior salt tolerance.

### 2.2 Salt stress treatment and experimental design

Salt stress was simulated using sodium chloride (NaCl). Four concentration gradients were established, such as 0 mM (control), 50 mM (mild stress), 100 mM (moderate stress), and 150 mM (severe stress) (Fellahi et al., 2024). Among these, 100 mM NaCl was commonly used as a standard screening concentration and was selected for physiological and transcriptional validation, balancing stress response and seedling viability, while 150 mM NaCl was used as a more stringent stress condition to maximize phenotypic discrimination among genotypes (Dadshani et al., 2019; Fellahi et al., 2024). The experiment followed a randomized complete block design with two independent replicates for each variety under all treatments. All treatments were conducted under uniform growth conditions, with controlled temperature, photoperiod, humidity, and culture duration to minimize environmental variation and ensure the reliability and comparability of phenotypic measurements.

### 2.3 Phenotypic measurement and salt tolerance index calculation

Phenotypic traits, including plant height (mm), root length (mm), germination rate, and salt tolerance index (STI), were measured at the germination and seedling stages. Each variety was evaluated in two independent experimental replicates at each NaCl concentration. For plant height and root length, five seedlings were measured within each replicate, and the mean value was used as the phenotypic estimate for that replicate to reduce measurement error and obtain stable trait estimates. Germination rate was calculated as the proportion of germinated seeds to the total number of seeds, and expressed as either a percentage (%) or a decimal. The salt tolerance index (STI) was calculated using a relative value method, defined as:


$$\text{STI}=\frac{T_{\text{stress}}}{T_{\text{control}}}$$

where T_stress_ represents the trait value under salt stress, and T_control_ represents the corresponding value under control conditions (Tao et al., 2021). In this study, STI values were pre-calculated and provided in the original dataset (see “Salt Tolerance Index” column in the Excel data), and were used as a key indicator in subsequent statistical analysis and comprehensive evaluations. To comprehensively assess salt tolerance, we integrated multiple phenotypic traits—including germination rate, plant height, root length, and the Salt Tolerance Index (STI)—into a multi-trait evaluation framework. STI was chosen as a core indicator because it reflects the relative performance of each genotype under salt stress compared to control conditions, capturing both stress-induced reduction and maintenance of growth. While individual traits reflect specific aspects of growth, STI provides an integrative measure that accounts for the overall stability of the genotype under stress, making it particularly suitable for inclusion in multi-trait ranking approaches such as TOPSIS. Using STI alongside germination, plant height, and root length ensures that the evaluation framework captures both specific trait sensitivity and holistic salt tolerance, thereby improving the reliability and biological relevance of the selection process.

### 2.4 Data processing and quality control

All raw phenotypic data were processed and archived in an Excel spreadsheet. The dataset included variety name, replicate number, salt treatment concentration, plant height, root length, germination rate, and salt tolerance index. Before statistical analysis, the data underwent quality control and standardization procedures. Variety names were standardized, and the units and numerical formats of salt concentrations were verified. Missing values and outliers were identified, flagged, and removed. For plant height and root length, the mean of repeated measurements for each variety under the same salt concentration and replicate condition was used as the final phenotypic value. The final dataset used for subsequent analysis contained 958 records, covering 120 varieties, four salt concentration gradients, and two independent replicates.

### 2.5 Phenotypic calculation and analysis workflow

**Figure 1:**
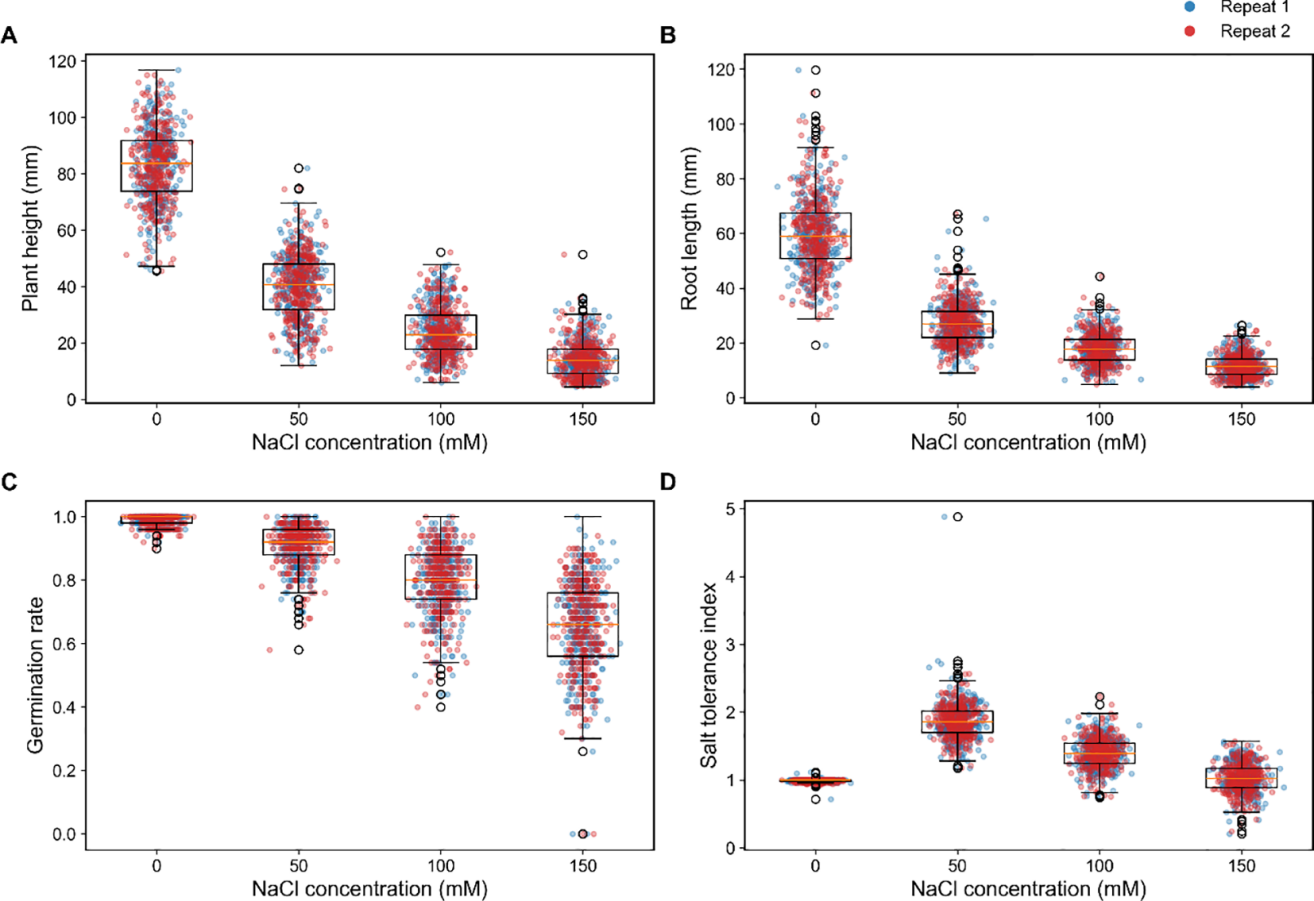
Distribution and repeatability of phenotypic traits under different salt concentration treatments. **(A)** Plant height (mm) distribution across salt levels. **(B)** Root length (mm) distribution across salt levels. **(C)** Germination rate distribution across salt levels. **(D)** Salt tolerance index distribution across salt levels. Two independent replicates (Repeat 1 and Repeat 2) are distinguished by color.

**Figure 2:**
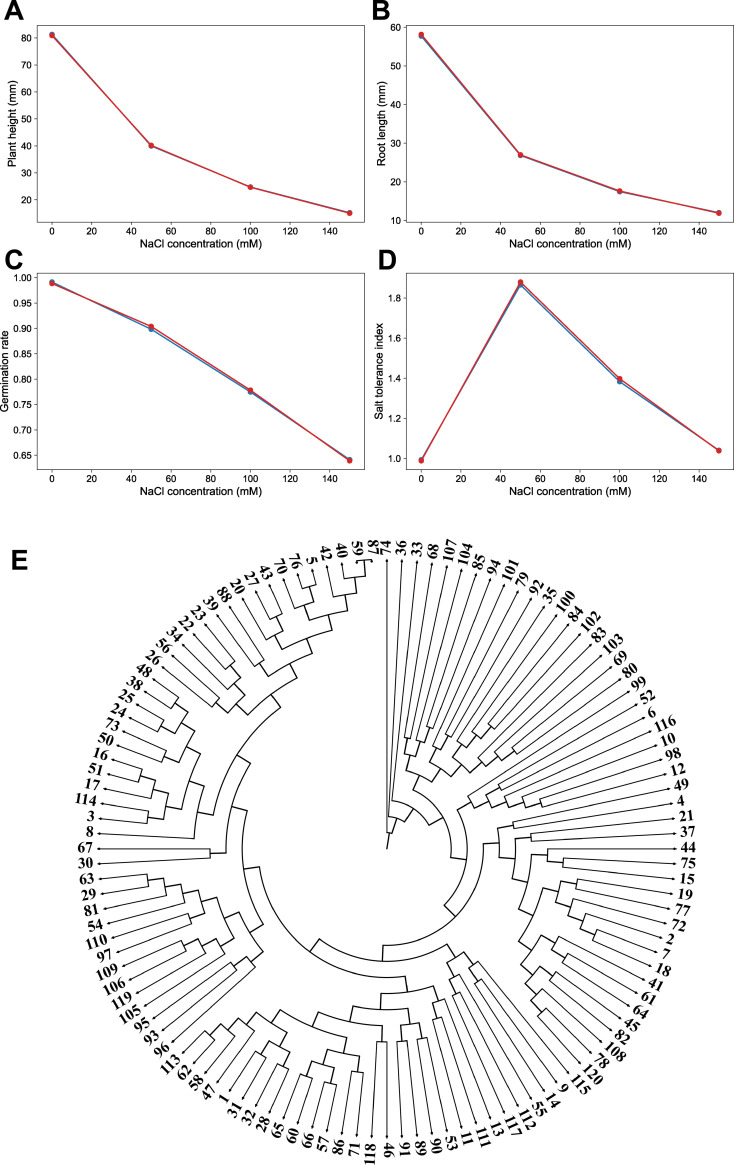
Dose–response analysis and phenotypic clustering of wheat varieties under salt stress. **(A)** Dose-response curve for plant height (mm). **(B)** Dose-response curve for root length (mm). **(C)** Dose-response curve for germination rate. **(D)** Dose-response curve for salt tolerance index. Two independent replicates are distinguished by color. **(E)** Phenotypic clustering tree of 120 wheat accessions based on multi-trait responses to salt stress (circular layout). The phenotypic matrix, including plant height, root length, germination rate, and STI across different salt concentration, was Z-score standardized. Euclidean distance was used to calculate dissimilarity among accessions, and hierarchical clustering was performed using the average linkage (UPGMA) method. The resulting tree was generated in Newick format. K-means clustering (K = 3) was applied to classify the accessions.

**Figure 3:**
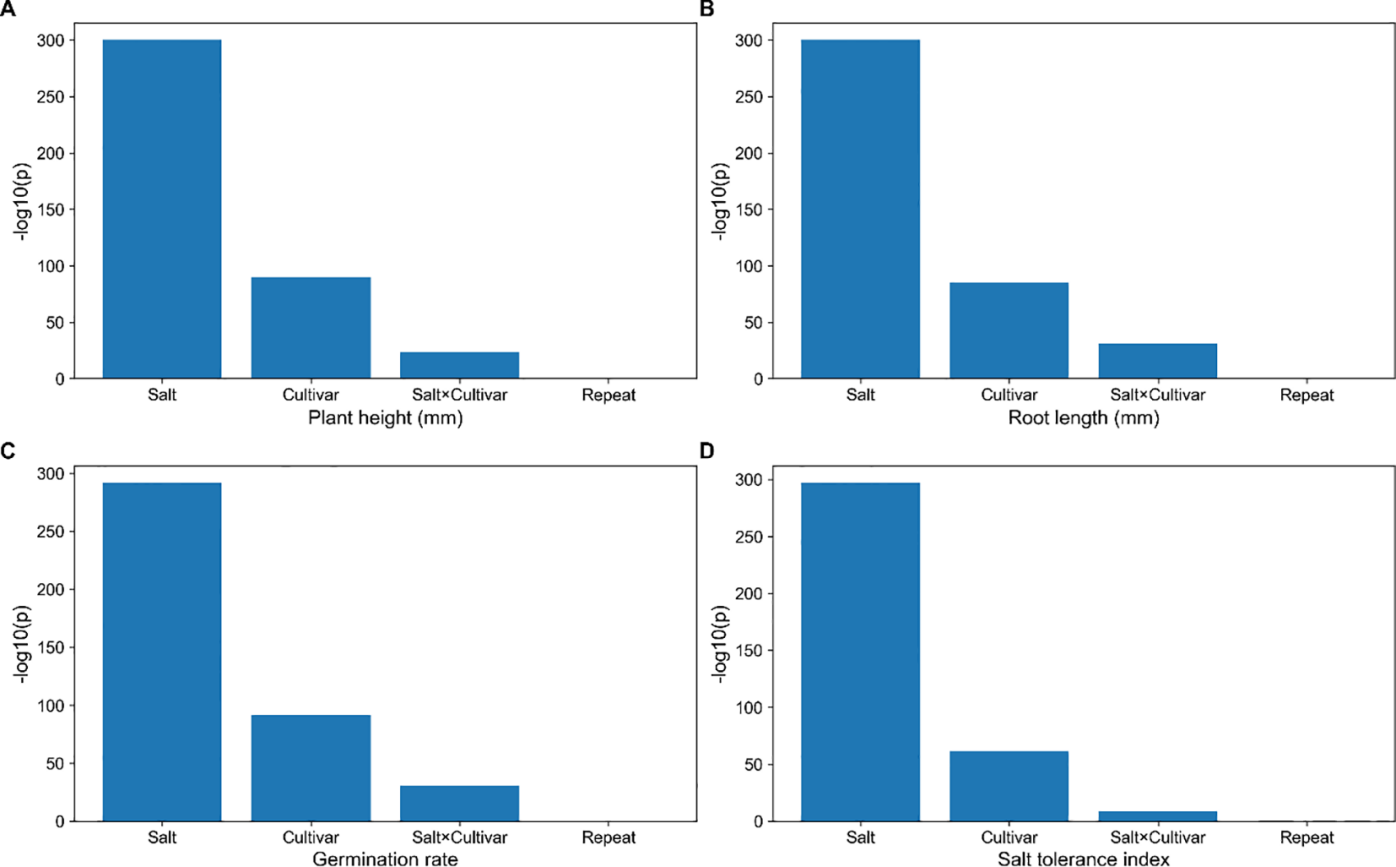
Analysis of variance (ANOVA) for key phenotypic traits under salt stress. **(A)** Plant height (mm); **(B)** Root length (mm); **(C)** Germination rate; **(D)** Salt tolerance index (STI). Significance of the effects of Salt concentration, Cultivar (genotype), their interaction, and Replicate are shown for each trait.

**Figure 4:**
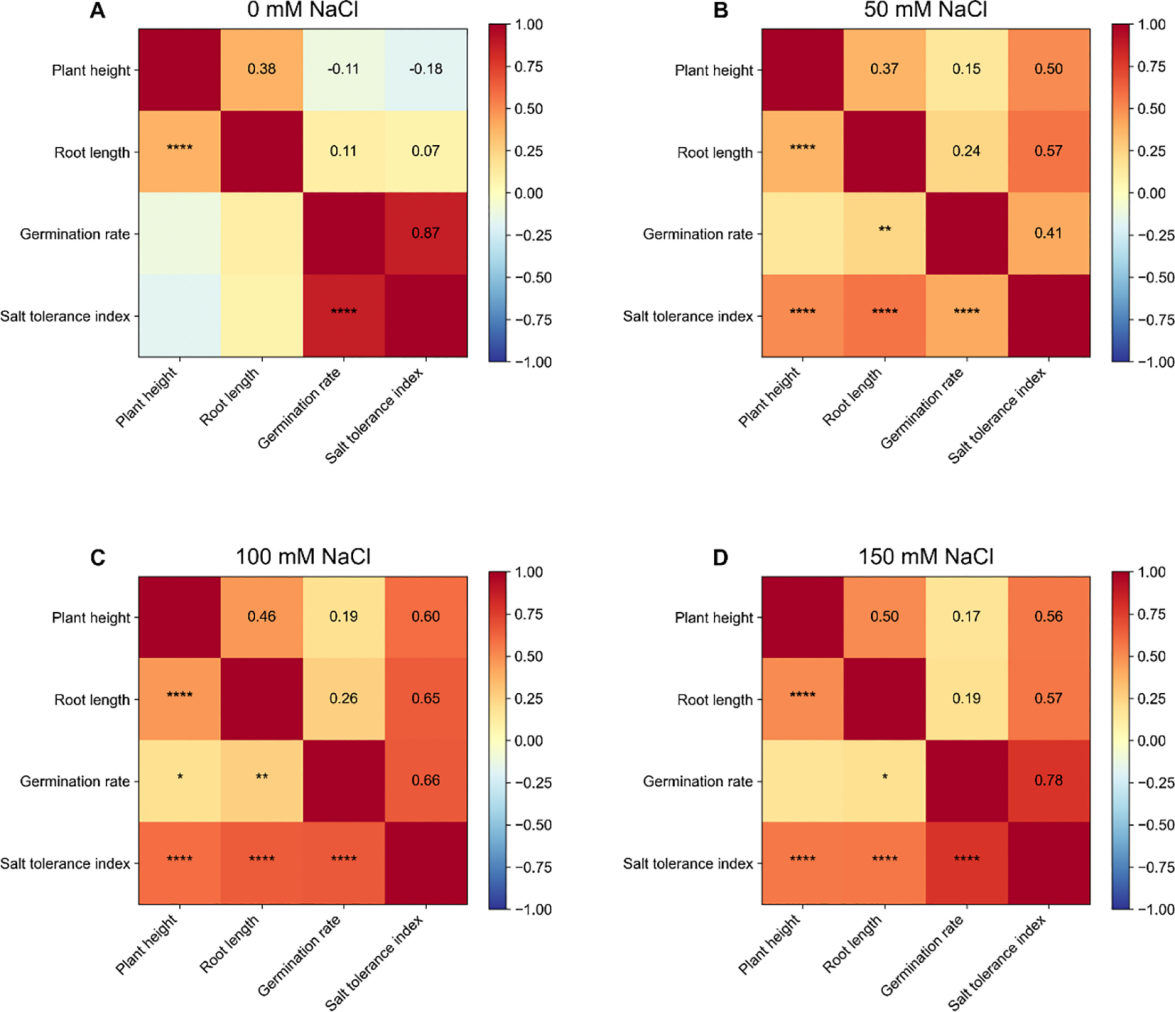
Pearson correlation analysis between phenotypic traits under different NaCl concentrations. **(A)** Correlation heatmap at 0 mM. **(B)** Correlation heatmap at 50 mM. **(C)** Correlation heatmap at 100 mM. **(D)** Correlation heatmap at 150 mM. In each heatmap, the upper triangle shows the correlation coefficient (r), and the lower triangle indicates significance levels using asterisks (*p<0.05, **p<0.01, ***p<0.001, ****p<0.0001).

**Figure 5:**
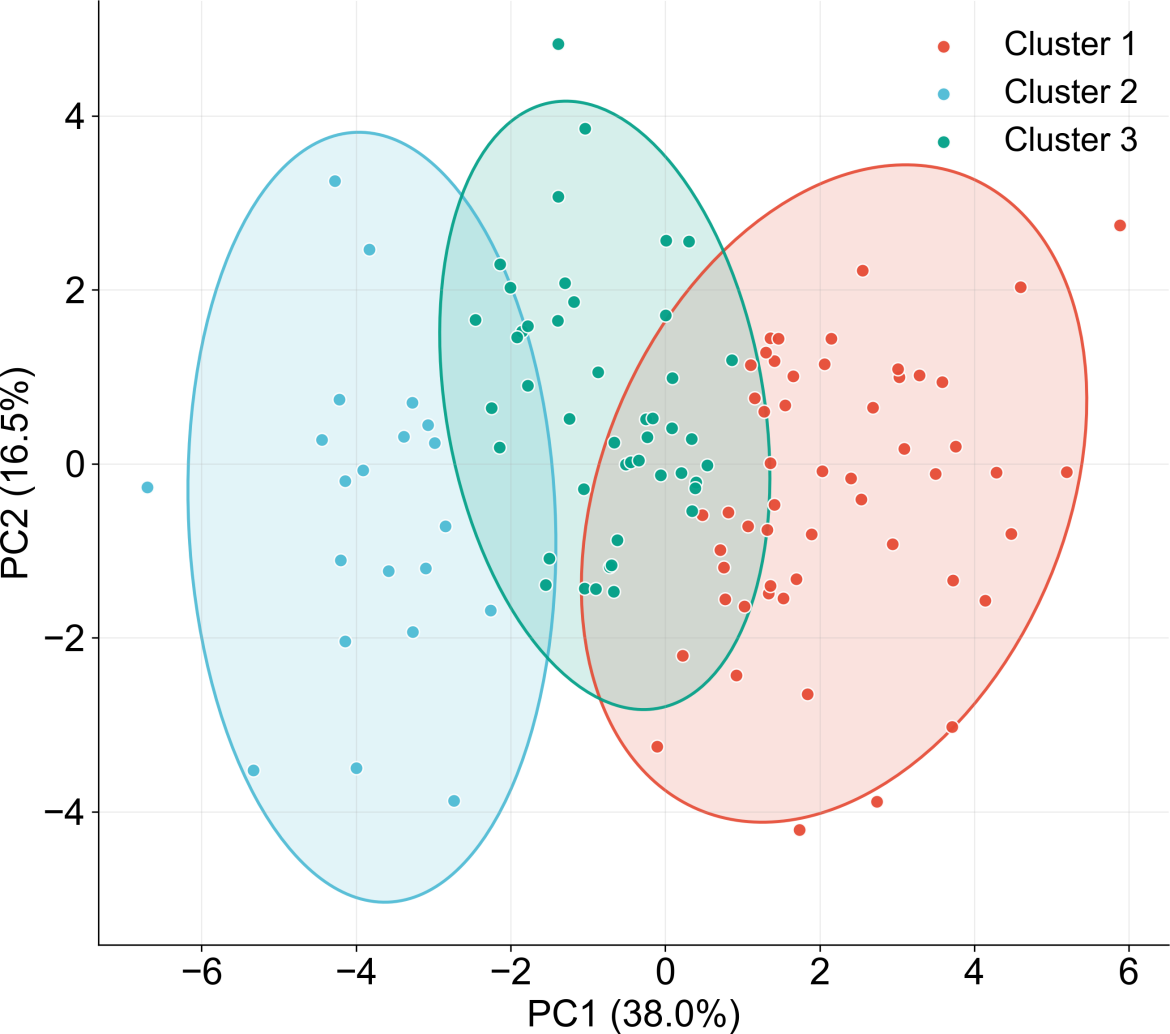
Principal component analysis (PCA) of standardized phenotypic traits. PC1 and PC2 capture the main variation patterns of the population. Each color represents a distinct cluster, illustrating stable multidimensional phenotypic patterns across salt treatments.

**Figure 6:**
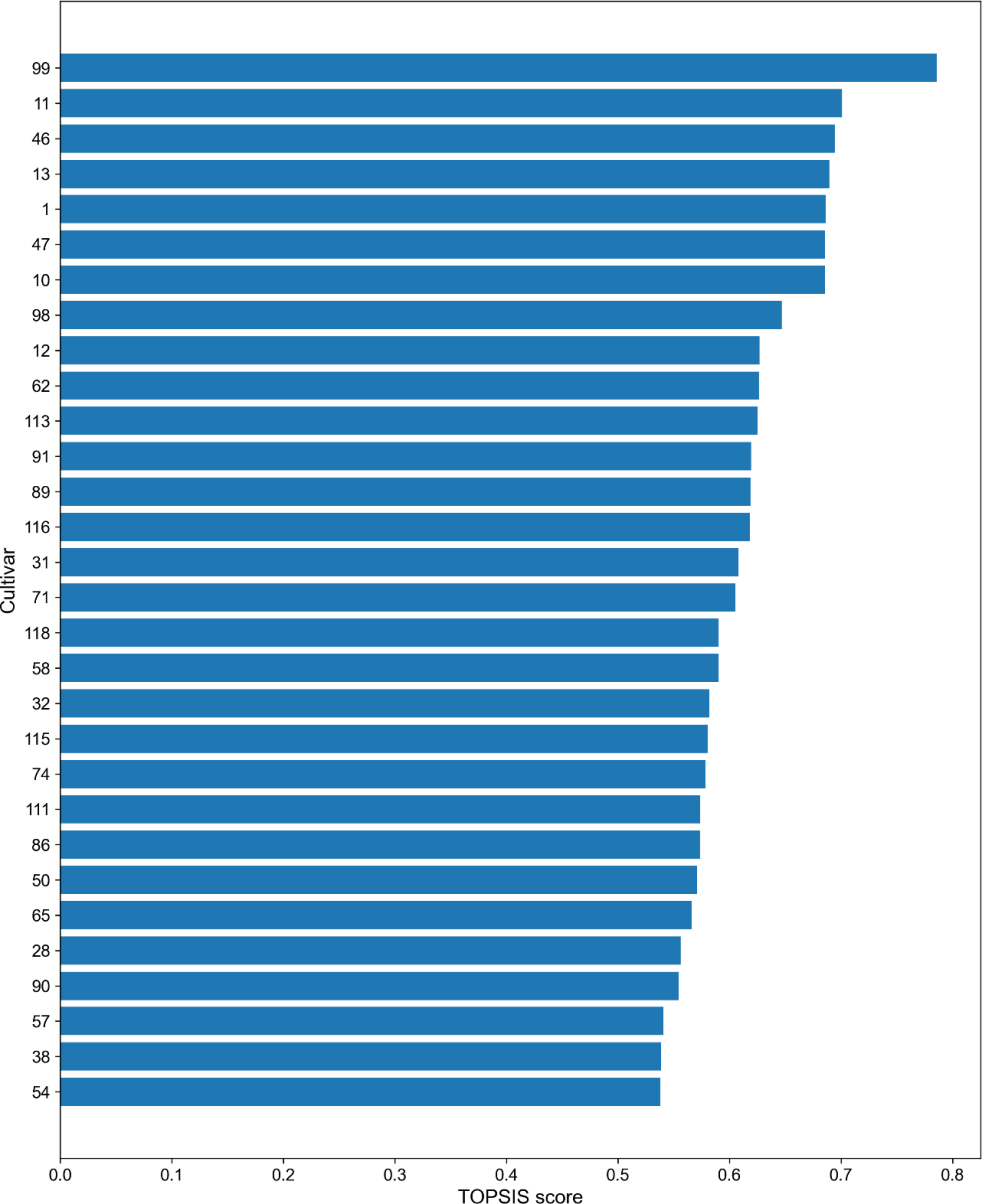
TOPSIS-based ranking of wheat varieties for salt tolerance. The 30 varieties with the highest TOPSIS scores are shown, representing the strongest salt-tolerant germplasm candidates under multi-trait and multi-salt gradient evaluation.

To systematically characterize phenotypic variation patterns under different salt concentration gradients, analyze trait correlations, reveal phenotypic clustering characteristics, and screen candidate materials with superior salt tolerance, a comprehensive phenotypic analysis workflow was constructed. First, in the phenotypic distribution and replicate consistency analysis (**Figure 1**), scatter plots of plant height, root length, germination rate, and salt tolerance index under different salt concentrations were drawn. The two independent replicates were distinguished by color to assess replicate consistency and population phenotype dispersion. Next, dose-response analysis was performed (**Figures 2A–D**). The average phenotypic values of two replicates at each salt concentration were calculated, and response curves were plotted to illustrate the influence of salt stress intensity on different traits. Based on this, a phenotypic phylogenetic tree was constructed (**Figure 2E**). Data were organized into a wide table matrix (“variety × trait × salt concentration”), standardized, and Euclidean distances were calculated. Hierarchical clustering using average linkage was applied to construct a phylogenetic tree, which was output in Newick format and visualized using ggtree (Yu et al., 2018; Paradis and Schliep, 2019). To evaluate the contributions of salt stress and genetic background to phenotypic variation (**Figure 3**), multivariate analysis of variance (ANOVA) was performed for each trait to test the significance of salt concentration, variety, replicates, and the salt concentration × variety interaction (Masarmi et al., 2023). Correlation analysis (**Figure 4**) was then conducted by calculating Pearson correlation coefficients under a representative salt concentration (preferably 100 mM), which were displayed as a correlation heatmap (Singh and Singh, 2024). The upper triangle shows the correlation coefficient (r), and the lower triangle indicates significance with asterisks (*p<0.05, **p<0.01, ***p<0.001, and ****p<0.0001). Further, PCA was performed on the standardized wide-table matrix to extract PC1 and PC2, highlighting the main variation patterns of the population (**Figure 5**). K-means clustering was used to explore phenotypic grouping. K = 3 was retained as a biologically interpretable solution consistent with PCA and hierarchical clustering, rather than being presented as a statistical optimum. Finally, multi-trait evaluation and ranking were conducted using the Technique for Order Preference by Similarity to Ideal Solution (TOPSIS) method (**Figure 6**). TOPSIS was applied to integrate multiple phenotypic traits, including plant height, root length, germination rate, and salt tolerance index (STI), into a single composite score for each variety. A higher TOPSIS score indicates a variety’s overall performance is closer to the ideal salt-tolerant phenotype across all salt concentrations, enabling robust identification of superior germplasm.

### 2.6 Software and statistical analysis environment

All data processing, statistical analysis, and visualization were performed under a Windows 10/11 operating system. The Python environment used was Python 3.12, with key third-party libraries including pandas, numpy, matplotlib, scipy, statsmodels, scikit-learn, and pillow (specific version information can be exported via pip freeze) (Fehler et al., 2023; Liang et al., 2024). Phylogenetic tree visualization was performed in R, (version 4.2 or higher recommended), using packages such as ggtree, ape, ggplot2, and dplyr (Paradis and Schliep, 2019). If the analysis environment lacked an Rscript command-line interface, the phylogenetic tree plots could be generated manually by running a script (e.g., draw_tree_Rscript.R) in RStudio to ensure reproducibility.

### 2.7 Reproducibility and data output

All data processing, statistical analysis, and figure generation in this study were automated using scripts. Running the main program (main.py) produces **Figures 1**–**6** and the associated tables in a single execution. For each figure, the output directory contains: the data file used for plotting (plot_data), the corresponding independent plotting script (code), and a high-resolution image file (PNG/PDF, default 300 dpi). This workflow ensures that the analysis is fully reproducible and that all results can be independently verified.

### 2.8 Physiological indicators and ion content determination

Based on the TOPSIS screening results, the salt-tolerant variety “99” (highest score) and the salt-sensitive variety “101” (lowest score) were selected as extreme materials for in-depth physiological mechanism analysis. Seedlings were grown to the three-leaf stage and then subjected to 100 mM NaCl stress treatment, with 0 mM NaCl as the control (CK). After 7 days of treatment, root tissues of both varieties were collected, immediately flash-frozen in liquid nitrogen, and stored at -80°C for further analysis.

Root physiological indicators included the activities of superoxide dismutase (SOD), peroxidase (POD), and catalase (CAT), as well as the contents of malondialdehyde (MDA) and free proline (Pro) (Gill and Tuteja, 2010; Zhao et al., 2022). All indicators were extracted using commercial kits from Solarbio Life Sciences, Beijing, China, and measured according to the manufacturer’s instructions using a UV-Vis spectrophotometer. Root Na+ and K+ contents were determined using a flame photometer (Alghabari and Shah, 2025). Root samples were first blanched at 105°C, dried to constant weight at 80°C, ground into powder, and digested with concentrated nitric acid-H_2_O_2_. After adjusting volume, ion concentration in the filtrate was measured, and the K^+^/Na^+^ ratio was calculated.

### 2.9 Multigene qRT-PCR expression analysis

Based on extreme phenotypic grouping, five salt-tolerant groups (1, 11, 13, 46, 99) and five sensitive varieties (35, 100, 101, 103, 104) were selected for root tissue sampling under 0 mM (control) and 100 mM NaCl treatment conditions. Total RNA was extracted from the root samples, and genomic DNA contamination was removed prior to cDNA synthesis via reverse transcription. Quantitative real-time PCR (qRT-PCR) was performed using the SYBR Green system on a real-time quantitative PCR instrument. *TaActin* was used as the internal control gene (Prasad et al., 2020). Relative expression levels were calculated using the 2^-ΔΔCt^ method, and primer sequences are listed in **Supplementary Table S1** (Livak and Schmittgen, 2001). Each sample was measured in three technical replicates per biological replicate to ensure accuracy and reproducibility.

## 3 Results

### 3.1 Overall distribution and repeatability of phenotypic traits under different salt concentrations

In the 120 wheat varieties evaluated, four key phenotypic traits, plant height, root length, germination rate, and salt tolerance index (STI), were systematically analyzed across four NaCl concentration gradients (0, 50, 100, and 150 mM). Overall, trait distributions differed markedly among different salt treatments, with the range of variation increasing progressively with salt concentration (**Figures 1A–D**). Under control condition (0 mM), plant height and root length exhibited relatively narrow distributions, and germination rate remained consistently high. With increasing salinity all three traits shifted towards lower values, accompanied by a substantial increase in dispersion, indicating that salt stress inhibited seedling growth and amplified phenotypic variability among genotypes. Notably, consistent trends were observed across both independent replicates (Repeat 1 and Repeat 2), and the overall distribution between replicates showed a high degree of overlap (**Figure 1**), demonstrating strong reproducibility and reliability of the experimental data. Under severe stress (150 mM NaCl), phenotypic divergence among varieties was most pronounced. While some genotypes maintained relatively high plant height and root length, others exhibited markedly reduced values or extreme phenotypes, suggesting that this concentration provides strong discriminatory power for identifying salt-sensitive materials.

### 3.2 Salt stress dose–response analysis reveals trait-specific sensitivity differences

To further characterize trait-specific responses across salt gradients, dose-response analysis were performed using the mean values of each trait at each NaCl concentration, and response curves were generated based on two independent replicates (**Figures 2A–D**). Overall, increasing NaCl concentration led to a progressive decline in plant height (**Figure 2A**), root length (**Figure 2B**), and germination rate (**Figure 2C**) with the reduction becoming more pronounced in the 100–150 mM range. This indicates that moderate-to-high salinity exerts a stronger inhibitory effect on germination and early seedling growth. Trait-specific sensitivity to salt stress was also evident. Plant height and root length declined even under mild stress (50 mM NaCl), whereas the germination rate remained stable at low salinity and only decrease substantially under higher salt concentrations. As a composite indicator, the salt tolerance index (STI) revealed pronounced genotypic differentiation with increasing salinity (**Figure 2D**): varieties maintained high STI values, whereas other exhibited a sharp decline beyond 100 mM NaCl. The response patterns were highly consistent between the two replicates, confirming the robustness and reproducibility of the dataset. Collectively, these results indicate that 100 mM NaCl effectively captures population-level variation and suitable for initial screening, whereas 150 mM NaCl provides stronger phenotypic discrimination and is more appropriate for stringent identification of salt-tolerant materials (Fellahi et al., 2024).

To comprehensively assess phenotypic similarity among the 120 wheat varieties across multiple salt gradients, a phenotypic distance matrix was constructed based on the mean values of four traits under 0, 50, 100, and 150 mM NaCl conditions. Hierarchical clustering was then performed to generate a phenotypic clustering tree (**Figure 2E**). The resulting tree exhibited branching patterns, indicating that the varieties could be grouped into distinct clusters with stable and differentiable multi-trait response profiles under salt stress. Specifically, a subset of varieties clustered together due to their ability to maintain relatively high plant height, root length, and germination rate across salt treatments, accompanied by consistently high salt tolerance index (STI) values, reflecting a salt-tolerant phenotype. In contrast, another group formed separate cluster characterized by pronounced growth inhibition under high salinity, with substantial reductions in multiple traits, indicative of salt-sensitive responses. Additionally, several varieties were positioned between these two extremes, displaying intermediate responses. Overall, the clustering patterns were highly consistent with the trends observed in the dose-response analysis (**Figures 2A–D**), further the existence of clear population-level differentiation in multi-trait responses. These results provide robust phenotypic evidence for the identification of extreme salt-tolerant and sensitive materials and support subsequent genetic and mechanistic analysis.

### 3.3 Analysis of variance reveals significant effects of salt concentration, genotype, and their interactions on phenotypic traits

To quantify the contribution of experimental factors to phenotypic variation, analysis of variance (ANOVA) was conducted for plant height, root length, germination rate, and salt tolerance index (STI). The significance of each factor was expressed as -log10(p) (**Figures 3A–D**). The results showed that both salt concentration (Salt) and genotype (Cultivar) had significant effects on all traits (p < 0.001), indicating that salinity acts as a major environmental driver seedling performance, while substantial genetic variation exists among the tested varieties. In addition to these main effects, the salt × cultivar interaction was also significant for multiple traits, demonstrating the presence of pronounced genotype × environment (G×E) interactions. This suggests that differences are not only reflected in overall trait performance but also in their differential response patterns and capacity to maintain growth under salt stress. These findings are consistent with the patterns observed in dose-response analysis and clustering results, further supporting the existence of distinct phenotypic response types among varieties under salinity stress. In contrast, the effect of the replicate factors were generally weak or non-significant, indicating high consistency between independent experiments, low experimental error, and overall robustness of the dataset.

### 3.4 Correlation analysis reveals strong associations between salt tolerance index and growth-related traits

To investigate the relationship among key phenotypic traits, Pearson correlation coefficients were calculated for the four core indicators under representative salt stress conditions (primarily 100 mM NaCl, or 150 mM where necessary). Correlation patterns were visualized using heatmaps, with correlation coefficients (r) displayed in the upper triangle and corresponding significance level indicated in the lower triangle (**Figure 4**). The results showed that plant height and root length were consistently and significantly positively correlated (r > 0, p < 0.05), indicating coordinated regulation between the aboveground and belowground growth under salt stress. The salt tolerance index (STI) exhibited moderate to strong positive correlations with plant height, root length, and germination rate, suggesting that STI effectively integrates multi-trait performance and reflect the relative ability of genotypes to maintain growth and germination under saline conditions. In contrast, the correlation between germination rate and growth-related traits (plant height and root length) were generally weaker, implying partial independence salt stress responses at the germination stage and subsequent seedling growth stages. These findings highlight the limitations of relying on a single trait evaluation for salt-tolerance screening and emphasize the importance multi-trait integration. Overall, the correlation analysis supports the use of STI as a robust composite indicator, in combination with key growth traits, for comprehensive assessment of salt tolerance in wheat germplasm (Tao et al., 2021).

### 3.5 Principal component analysis and clustering reveal stable multidimensional phenotypic structures among varieties

To further capture the major patterns of variations in the multidimensional phenotypic dataset, the data from 120 wheat varieties were standardized based on a wide-format feature matrix comprising “four traits × four salt concentrations,” followed by principal component analysis (PCA). K-means clustering was subsequently performed using the principal component scores (**Figure 5**), with K = 3 retained as a biologically interpretable grouping consistent with PCA and hierarchical clustering, rather than being presented as a statistical absolute. The PCA results indicated that the first two principal components (PC1 and PC2) explained a substantial proportion of the total variation, accounting for approximately 38% and 16.5% respectively. this suggests that a limited number of components effectively summarize the major phenotypic differences within the population. In the PC1-PC2 space, the varieties exhibited clear distribution patterns and distinct grouping tendencies. K-means clustering further divided the population into three major clusters (Cluster 1-3) (**Figure 5**), each characterized by different overall phenotypic performance under salt stress. For instance, cluster with higher overall PC1 scores, were generally associated with improved maintenance of growth-related traits, indicative of stronger salt tolerance, whereas clusters located in the negative region in the PC space exhibited reduced trait performance, reflecting greater sensitivity to salinity. Importantly, the clustering patterns identified by PCA were highly consistent with the phenotypic relationships observed in the hierarchical clustering tree (**Figure 2E****),** reinforcing the existence of stable and structured phenotypic differentiation among the varieties. These results provide a robust, data-driven framework for classifying germplasm, defining salt tolerance levels, and selecting candidate materials based on integrated phenotypic characteristics.

### 3.6 Identification of salt-tolerant varieties using TOPSIS multi-indicator comprehensive evaluation

Given the differential responses of individual traits to salt stress and the limitations of single trait evaluation, the TOPSIS (Technique for Order Preference by Similarity to Ideal Solution) method was employed to perform a comprehensive, multi-indicator assessment of salt tolerance across the 120 wheat varieties (**Figure 6**). This approach integrates multiple phenotypic traits to generate a composite score for each variety. Higher scores indicate varieties closer to the ideal multi-trait salt-tolerant phenotype. Using these scores, the top- and bottom-ranked varieties were selected as extreme salt-tolerant and salt-sensitive lines for further physiological and molecular validation, providing a comprehensive evaluation framework. The results revealed substantial variation in TOPSIS scores among varieties, with a clear separation between high- and low performing groups (**Figure 6**). The top-ranked varieties (e.g., Top 30) consistently maintained higher plant height, root length, and germination rate under moderate-to-high salt stress, along with elevated STI values, indicating strong salt tolerance. In contrast, varieties with lower scores generally showed significantly inhibited growth under strong salt treatment conditions, accompanied by a decreasing trend in salt tolerance index. Overall, the TOPSIS-based evaluation results provide a robust and quantitative framework for multi-trait information, enabling reliable identification of superior salt-tolerant germplasm. In addition, it defines a representative subset of extreme materials that can serve as valuable resources for downstream genetic analysis, including genotyping, QTL mapping, or association studies.

### 3.7 Molecular validation of functionally relevant salt-responsive genes

**Figure 7:**
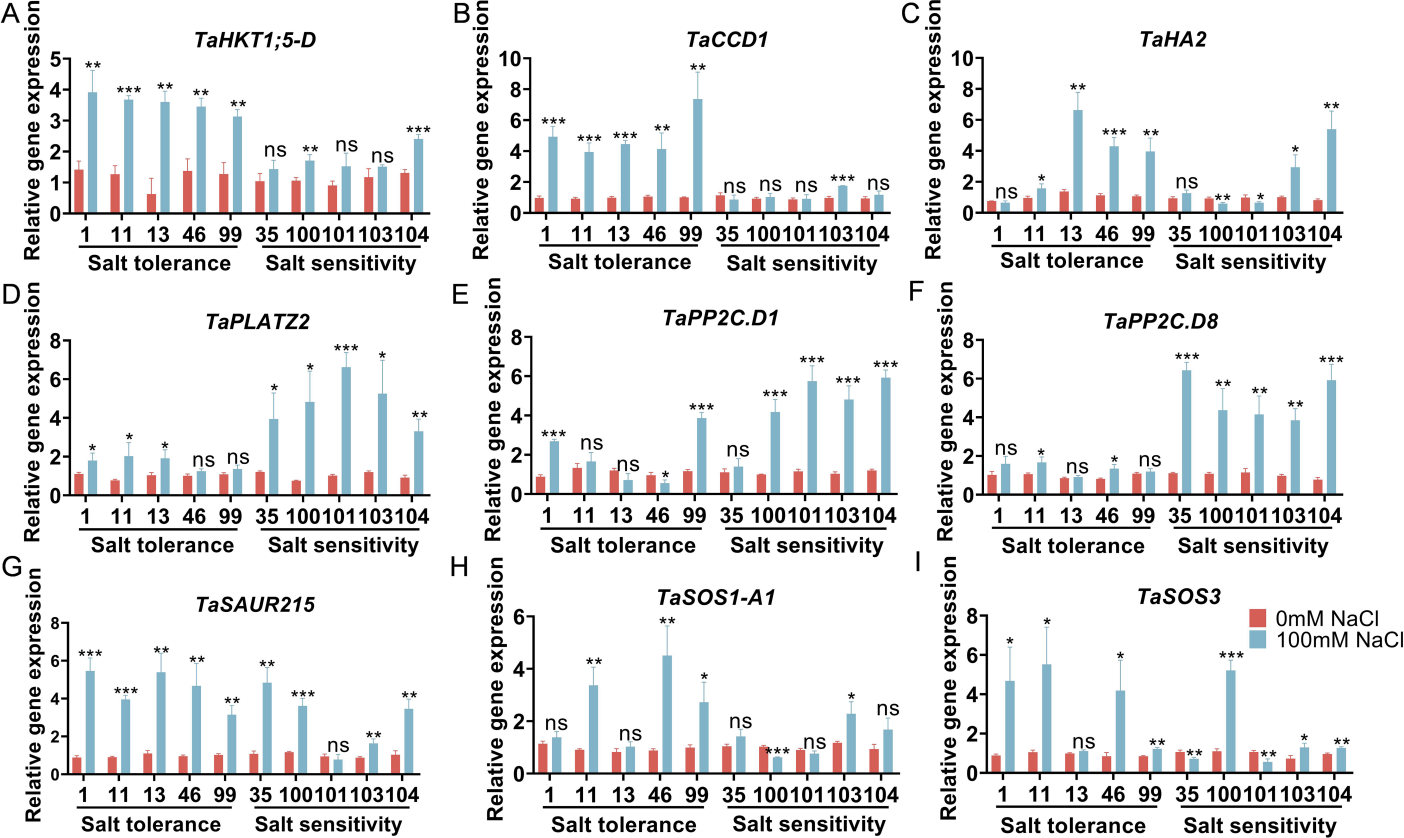
Relative expression levels of nine candidate genes in the roots of salt-tolerant and salt-sensitive wheat materials under salt stress. Expression was evaluated in 10 varieties; 5 from the TOPSIS-ranked salt-tolerant group (1, 11, 13, 46, 99) and 5 from the salt-sensitive group (35, 100, 101, 103, 104). Relative expression levels of *TaHKT1;5-D* **(A)**, *TaCCD1* **(B)**, *TaHA2* **(C)**, *TaplatZ2* **(D)**, *TaPP2C.D1* **(E)**, *TaPP2C.D8* **(F)**, *TaSAUR215* **(G)**, *TaSOS1-A1* **(H)**, and *TaSOS3* **(I)** was evaluated in root tissues under 0 mM NaCl (control) and 100 mM NaCl (salt treatment) conditions. Bars represents the mean ± SD (n = 3 biological replicate). Statistical significance indicates differences between the same variety under 100 mM NaCl versus 0 mM NaCl: *p* < 0.05, *p* < 0.01, *p* < 0.001; *p* ≥ 0.05; ns indicates non-significant differences. *p < 0.05, **p < 0.01, *p < 0.001.

To validate the molecular basis underlying the phenotypic screening results, we evaluated the expression patterns of the selected genes in the extreme salt-tolerant (99) and salt-sensitive (101) varieties under control and multi-gradient salt stress conditions (**Figure 7**). The selected genes were chosen based on their established roles in salt stress adaptation. For instance, TaHKT1;5-D functions as a Na^+^ transporter critical for maintaining root ion homeostasis by retrieving Na^+^ from the xylem, reducing shoot toxicity (Byrt et al., 2014). TaSOS1-A1 participates in Na^+^ efflux and long-distance Na^+^ transport, forming part of the SOS signaling pathway (Gao et al., 2023). TaHA2 regulates transmembrane proton gradients essential for ion compartmentalization (Cui et al., 2023). TaCCD1 and TaSAUR215 are involved in adaptive responses, including phytohormone signaling and growth modulation under stress (Cui et al., 2023). Conversely, TaPP2C.D1, TaPP2C.D8, and TaPLATZ2 function in upstream signal transduction and transcriptional regulation (Wei et al., 2025). Under control conditions, basal expression levels of most genes showed no significant differences between the two groups (P ≥ 0.05), indicating comparable initial transcriptional states. In contrast, under salt stress, the salt-tolerant group exhibited a coordinated induction pattern characterized by enhanced ion homeostasis and adaptive response mechanisms. Specifically, *TaHKT1;5-D* was significantly upregulated in all tolerant varieties (P < 0.01 or P < 0.001) (Xu et al., 2020), while *TaCCD1* and *TaSAUR215* were also significantly upregulated in the salt-tolerant varieties (TaCCD1 mostly P < 0.001; *TaSAUR215* P < 0.01-0.001). These results suggested that salt-tolerant genotypes effectively activate ion transport, homeostasis maintenance and adaptive transcriptional responses under salt stress (Cui et al., 2023). In addition, *TaHA2* expression was significantly upregulated in all salt-tolerant varieties except for variety 1 (P < 0.05–0.001) indicating enhanced regulation of transmembrane electrochemical gradients, which likely contributes to improved ion homeostasis under salt stress (**Figures 7A–C, G**).

In contrast, the salt-sensitive group exhibited a distinct transcriptional response pattern, characterized by strong activation of signal transduction and transcriptional regulatory module, but relatively unstable regulation of core ion homeostasis mechanisms. Specifically,*TaPP2C.D1* and *TaPP2C.D8* were significantly upregulated in most or all varieties in the sensitive varieties (*TaPP2C.D1*: all except 35, P < 0.001; *TaPP2C.D8*: all 5 varieties, P < 0.01 or P < 0.001) (Cui et al., 2023). Similarly, *TaPLATZ2* was consistently induced in the sensitive group, with particularly strong upregulation observed in variety 101 (P < 0.001) (**Figures 7D–F**) (Wei et al., 2025). However, genes directly involved in ion homeostasis showed significant instability in the salt-sensitive group. *TaHKT1;5-D* was significantly upregulated only in varieties 100 (P < 0.01) and 104 (P < 0.001), while remaining non-significant in the other genotypes (P ≥ 0.05). Likewise, *TaSOS1-A1* showed no significant induction in most strains in the sensitive varieties and was even significantly downregulated at 100 (P < 0.001), indicating impaired or was mis-regulated activation key component SOS pathway (Gao et al., 2023). Collectively, these results suggest that salt-tolerant genotypes establish coordinated regulatory network integrating ion homeostasis (HKT/SOS/HA2) and adaptive response pathways (CCD1/SAUR215). In contrast, salt-sensitive genotypes, despite strong activation of upstream regulatory modules such as PP2C/PLATZ2, exhibit insufficient or unstable activation of core homeostasis mechanisms, which likely underlies their reduced capacity to cope salt stress.

### 3.8 Physiological basis of salt tolerance in extreme varieties

**Figure 8:**
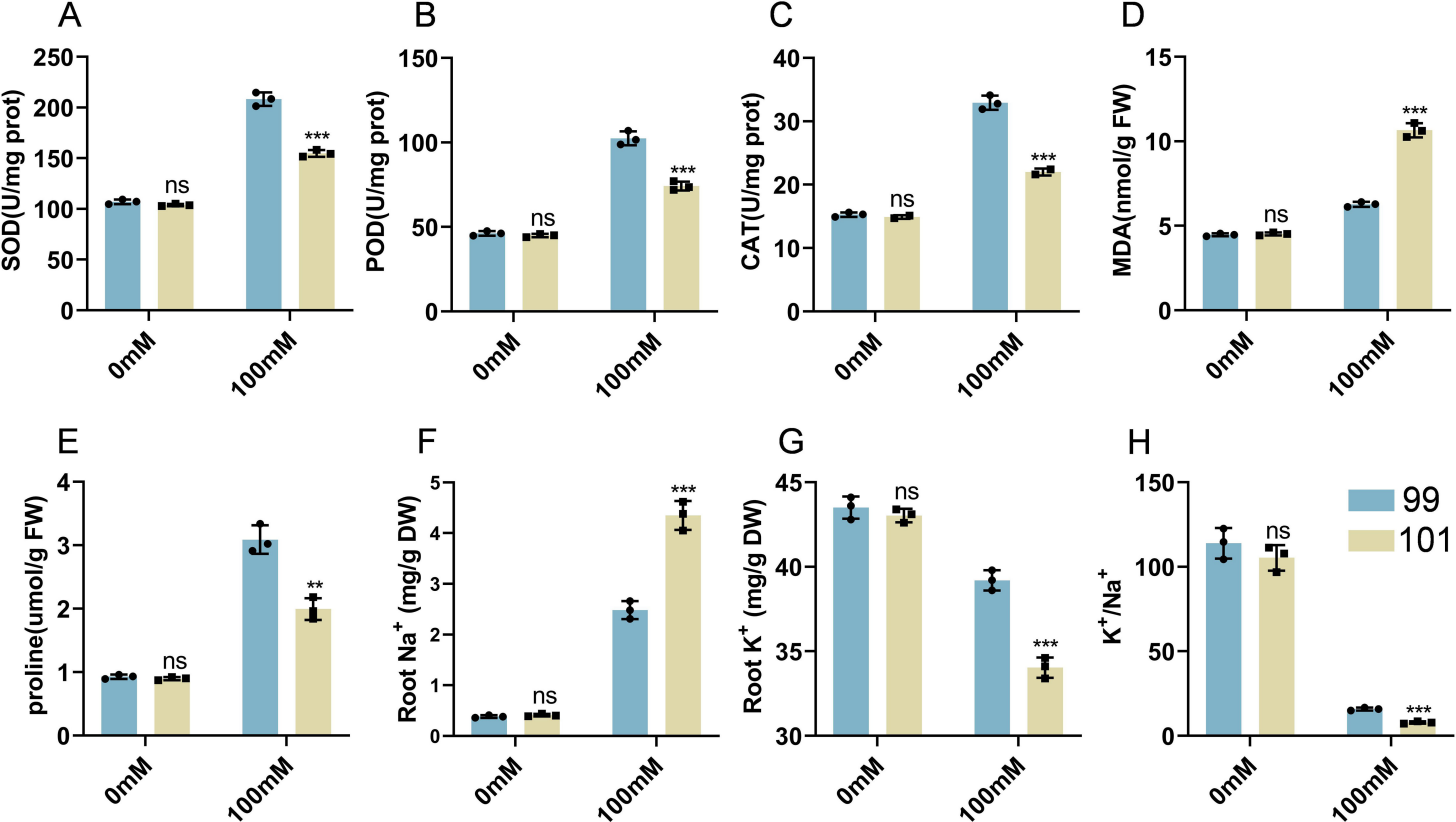
Root physiological indicators and Na^+^/K^+^ ion homeostasis in salt-tolerant and salt-sensitive materials under salt stress. Salt-tolerant variety 99 and salt-sensitive variety 101 were selected for analysis. Root tissues were collected under 0 mM NaCl (control) and 100 mM NaCl (salt treatment) conditions to measure: SOD activity **(A)**, POD activity **(B)**, CAT activity **(C)**, MDA content **(D)**, proline content **(E)**, Na^+^ content **(F)**, K^+^ content **(G)**, and K^+^/Na^+^ ratio **(H)**. Bars represent the mean ± SD (n = 3 biological replicates). Statistical significance indicates differences between control and salt treatment: P < 0.05, P < 0.01, P < 0.001; ns indicates non-significant differences (P ≥ 0.05). **p < 0.01, *p < 0.001.

To further validate the physiological basis of salt tolerance, root tissue of the salt-tolerant representative variety 99 and the salt-sensitive 101 were analyzed antioxidant capacity, membrane lipid peroxidation level, osmotic regulation, and ion homeostasis under control (0 mM) and salt (100 mM NaCl) conditions (**Figure 8**). Under control conditions, no significant differences in SOD, POD, and CAT activities, or in MDA and proline contents (all P ≥ 0.05), indicating that their basic physiological states were similar. Under salt treatment (100 mM NaCl), indicating comparable baseline physiological states. In contrast, under salt stress, the antioxidant system of variety 99 was markedly enhanced: SOD, POD, and CAT activities were all significantly higher than those in variety 101 (all P < 0.001), reflecting a stronger capacity for reactive oxygen species (ROS) scavenging. Consistently, MDA content was significantly higher in the sensitive variety 101 (P < 0.001), suggesting more severe lipid peroxidation damage and cellular damage. In terms of osmotic regulation, proline content increased in both varieties under salt stress; however, accumulation was significantly greater in variety 99 (P < 0.01), suggesting a more effective osmotic adjustment capacity. Ion analysis further revealed that, under 100 mM NaCl, the Na^+^ content in the roots of variety 101 was significantly higher than in variety 99 (P < 0.001), whereas K^+^ content was substantially higher in variety 99 (P < 0.001), consequently the K^+^/Na^+^ ratio was substantially higher in the tolerant variety (P < 0.001) (**Figure 8**). Importantly, the physiological and ion homeostasis measurements in representative varieties (99 and 101) corroborate the phenotypic screening results. The superior antioxidant enzyme activities, lower MDA, higher proline accumulation, and favorable Na^+^/K^+^ ratios in variety 99 correspond to its high TOPSIS score and robust seedling growth under multi-gradient salt stress. Conversely, the elevated Na^+^ accumulation, lower K^+^/Na^+^ ratio, and weaker antioxidant responses in variety 101 align with its poor performance in phenotypic screening. These results provide mechanistic validation that the extreme salt-tolerant and salt-sensitive phenotypes identified through multi-trait and multi-gradient analysis reflect genuine physiological differences in stress response, supporting the reliability of the screening approach.

### 3.9 Integrated multiscale analysis reveals robust phenotypic differentiation and enables reliable identification of salt-tolerant materials

Integrated analysis across phenotypic, statistical, and molecular/physiological levels consistently demonstrated that the 120 wheat varieties exhibited pronounced and distinguishable variation in salt tolerance under four NaCl gradients (0, 50, 100, and 150 mM) at both the germination and seedling stages. Descriptive statistics (**Figure 1**) and dose-response analysis (**Figure 2**) revealed that salinity significant inhibited key traits, including germination rate, plant height, and root length. Moreover, phenotypic divergence among varieties increased progressively with salt concentration, particularly under moderate-to-high salt levels (100–150 mM NaCl), indicating that elevated salinity enhances the expression of genetic variation and facilitates discrimination among varieties. Multivariate analysis further supported the existence of structured phenotypic differentiation. Hierarchical clustering, PCA, and K-means analysis based on multi-trait and muti-gradient data (**Figures 2E**, **5**) consistently identified stable and reproducible grouping patterns, suggesting that the salt tolerance responses among varieties are systematic and classifiable. ANOVA results (**Figure 3**) showed that salt concentration, genotype, and their interactions significantly affected phenotypic variation, highlighting both a strong genetic basis and pronounced genotype × environment (G×E) effects. In addition, correlation analysis (**Figure 4**) revealed coordinated relationships among traits and demonstrated that the Salt Tolerance Index (STI) is significantly associated with key growth parameters. This supports the use of STI as a robust integrative indicator for multi-trait performance and improving the reliability of salt tolerance assessment.

**Figure 9:**
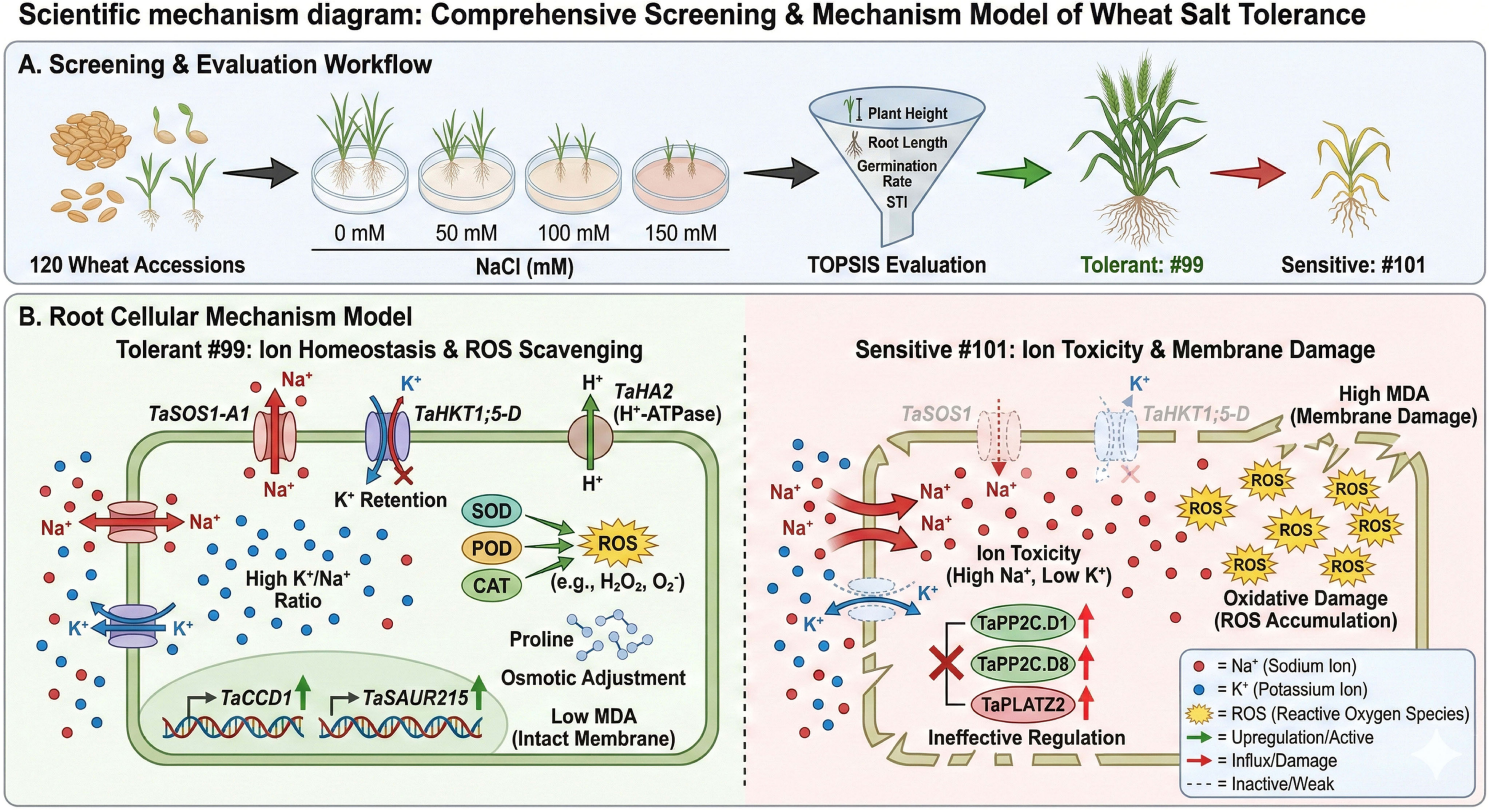
Schematic model of wheat salt tolerance: integrated screening workflow and underlying physiological/molecular mechanisms.

Based on these findings, the TOPSIS multi-index evaluation method (**Figure 6**) enabled quantitative ranking of salt tolerance across the 120 varieties and facilitated the identification of both highly tolerant and highly sensitive genotypes, providing well defined targets for subsequent validation. At the molecular level, qRT-PCR analysis of nine salt responsive genes in root tissue (**Figure 7**) showed that the salt-tolerant varieties exhibited coordinated and stable induction of genes related to ion homeostasis and adaptive response (e.g., *TaHKT1;5-D*) whereas salt-sensitive varieties showed predominant activation of signaling and transcriptional regulatory modules, but with unstable or insufficient activation of the core homeostasis pathways. These molecular patterns strongly support the phenotypic screening results. Consistently, physiological and ion homeostasis analysis (**Figure 8**) demonstrated clear differences between representative tolerant and sensitive materials. The salt-tolerant material displayed enhanced antioxidant capacity, lower membrane lipid peroxidation, and more effective osmotic adjustment, along with improved Na^+^ exclusion and K^+^ retention, resulting in a higher K^+^/Na^+^ ratio. These physiological traits further validate the stability and functionals basis of the salt-tolerant phenotype. Collectively, the multi-scale evidence—from multi-gradient phenotypic assessment and statistical modeling to TOPSIS-based ranking and molecular-physiological validation provides a coherent and mutually reinforcing framework. This integrative approach demonstrates that the established screening strategy can reliably identify wheat germplasm with excellent salt tolerance, thereby offering a solid foundation for subsequent gene discovery and breeding applications (**Figure 9**).

## 4 Discussion

Salt stress is a major limiting factor for wheat production, effecting multiple growth stages and traits. The result of this study highlights not only the variability in phenotypic responses among 120 wheat varieties but also the complex interaction between genotypes and stress intensity. In the following sections, we discuss how dose-dependent salt stress influences germination and seedling growth, how multi-trait evaluation can reveal robust salt-tolerant candidates, and the underlying molecular and physiological mechanisms that support these phenotypic observations.

### 4.1 Dose-dependent effects of salt stress on germination and seedling growth amplify phenotypic differences among wheat varieties

Salt stress is a major constraint on wheat germination and early seedling development, primarily through the combined effects of osmotic stress and ion toxicity (EL Sabagh et al., 2021). In this study, systematic evaluation of 120 wheat varieties across NaCl gradients (0, 50, 100, and 150 mM) demonstrated a clear dose-dependent decline in plant height, root length, germination rate, and STI, with the most pronounced reductions occurring under moderate-to-high salinity (100–150 mM) (**Figures 1**, **2**). Notably, 100 mM NaCl provided a robust, biologically stable stress suitable for downstream physiological and molecular validation, whereas 150 mM NaCl served primarily to distinguish extreme phenotypes among the varieties. These findings indicates that higher salt intensities not only impose stronger growth inhibition but also enhance the resolution of phenotypic differences among genotypes (Malipatil et al., 2025). Notably, phenotypic dispersion increased under moderate-to-high salt conditions, suggesting that severe stress acts as a “phenotypic differentiation amplifier” that exposes underlying genetic variation. This observation supports the widespread use of elevated salt concentrations in germplasm screening to improve discriminatory power (Fellahi et al., 2024; Malipatil et al., 2025). Importantly, the multi-gradient experimental design enables the characterization of the full response trajectory of each genotype, distinguishing between materials that are remain stable under mild stress but collapse beyond a threshold and those that maintain performance across a broad stress range (El-Hendawy et al., 2005; Tao et al., 2021). Such differentiation is particularly crucial for agriculture applications, where salinity level often vary spatially and temporally. Genotypes exhibiting stability across a wide range of salt intensities are therefore more desirable than those performing well under a single condition (Li et al., 2020; Wang et al., 2025a, 2025b). Overall, this study highlights the importance of dose-dependent evaluation in salt tolerance screening and at the phenotypic level and demonstrated that moderate-to-high salinity conditions substantially improve the efficiency and reliability of identifying superior germplasm by increasing phenotypic resolution without implying amplification of underlying genetic effects (**Figure 9**).

Different phenotypic traits reflect the distinct physiological processes and developmental stages affected by salt stress (Negrão et al., 2017). In this study, both plant height and root length decreased significantly with increasing salinity (**Figures 2A, B**), with root length generally exhibiting greater sensitivity. This is consistent with the direct exposure of roots to the saline conditions, where ion accumulation and osmotic stress primarily disrupt root meristem activity, cell division and elongation (Robin et al., 2016; Hao et al., 2023). Consequently, root length serves as a sensitive and discriminative trait for evaluating salt tolerance at the seedlings stage (Khan et al., 2022). In contrast, germination rate displayed a more stage-specific response pattern. Some genotypes maintained relatively high germination rates under low salinity, but exhibited pronounced inhibition during subsequent seedling growth, whereas others showed the opposite trend (Xu et al., 2024).This divergence suggests that the salt tolerance mechanisms during germination largely governed by processes such as endosperm mobilization, seed coat permeability, and early osmotic adjustment may be partially from those operating during seedling development, which rely more heavily ion homeostasis, antioxidant defense systems, and hormone regulation (Farooq et al., 2024; Manono, 2025). These findings highlight the limitations of single-trait screening approaches, as reliance on either or growth related traits alone may lead to biased evaluations (Tao et al., 2021; Dadrasi et al., 2024; Shi et al., 2025). Consistent with this, correlation analysis demonstrated that the salt tolerance index (STI) was significantly and positively associated with plant height, root length, and germination rate (**Figure 4**), indicating its capacity to integrate multi-trait responses and reflect the overall ability of genotypes to maintain performance under salt stress (Vineeth et al., 2026). Therefore, this study supports the use of STI as a core integrative indicator, complemented by key traits such as root length, plant height, and germination rate. This multi-dimensional evaluation framework captures both stage-specific and organ-specific responses, thereby enhancing the accuracy and robustness of salt tolerance screening.

### 4.2 Genotypic differences, salt × variety interactions, and multi-trait evaluation methods reveal stable phenotypic clustering of wheat under salt stress

Salt tolerance is a complex quantitative trait, with phenotypic differences arising not only from differences in stress intensity but also from genotypic background and genotype × environment (G×E) interactions (Chen et al., 2024; Ma et al., 2025a). in this study, ANOVA resulted showed that salt concentration, genotype, and their all interactions significantly contributed to trait variation (**Figure 3**), indicating the complexity of salt stress responses among different wheat varieties. These findings indicate that genotype differ not only in their baseline performance but also in their sensitivity and responses dynamics under increasing salinity. This interaction-driven variability explains the distinct response pattern observed among varieties, where some genotypes maintain stable performance under low salinity but exhibited rapidly decline beyond a threshold, whereas others display consistent performance across a wide range of salt conditions, reflecting more robust tolerance. Furthermore, phylogenetic trees, PCA, and cluster analyses constructed from multiple salt gradients and multiple traits revealed clear and reproducible cluster structures among the materials (**Figures 2E**, **5**). This suggests that salt tolerance response patterns are not randomly distributed but instead reflect coordinated “strategy-based” adaptations, whereby different groups of genotypes relay on distinct combinations of physiological mechanisms, such as ion homeostasis, osmotic regulation, or mitigating oxidative stress (Roy et al., 2014; Xiao and Zhou, 2023; Jha et al., n.d.). Importantly, these table grouping patterns enhance the interpretability of phenotypic screening and provide a valuable framework for downstream genetic analysis. For example, comparative analyses between salt-tolerant and sensitive groups, as well as investigations of extreme phenotypes, can facilitate the identification of key loci, regulatory pathways, and candidate genes associated with salt tolerance. In this way, phenotypic clustering can be effectively translated into mechanistic classification and actionable information for breeding programs.

Under multi-trait evaluation frameworks, reliance on single-trait threshold often fails to capture the complexity of salt tolerance and may lead to biased, such as identifying materials with superior in one trait but limited overall tolerance (Tabassum et al., 2021; Zhang et al., 2021; de Ocampo et al., 2025). In this study, the TOPSIS multi-indicator evaluation method was applied to integrate key traits including plant height, root length, germination rate, and STI into a unified composite score, enabling quantitative ranking of 120 wheat varieties (**Figure 6**). This approach provides a continuous and comparable measure of salt tolerance across genotypes. Compared with traditional screening methods, TOPSIS offers several advantages: it simultaneously incorporates multiple traits, reduces bias associated with single indicators, and facilitates efficient identification of extreme phenotypes representing both superior and inferior performance. These features make it particularly suitable for large scale germplasm evaluation and subsequent mechanistic or genetic analyses (Oliveira et al., 2022; Pandey et al., 2023). In addition to multi-trait integration, the selection of appropriate salt concentration is critical for balancing screening sensitivity and reliability. Moderate stress (100 mM NaCl) is effective for initial screening, allowing retention of a broad range of candidate materials, whereas higher stress (150 mM NaCl) provides stronger discriminatory power for identifying highly tolerant genotypes (Fellahi et al., 2024). Consistent with this, our study showed increased trait inhibition and phenotypic dispersion in the 100–150 mM range (**Figures 1**, **2**), supporting the use of a tiered screening strategy. The combined framework of “100 mM initial screening + 150 mM stringent screening” effectively balances efficiency and accuracy: moderate stress avoids widespread phenotypic collapse and preserves variability, while severe stress ensure that the selected materials exhibit stable performance under high salinity. This strategy aligns with breeding objective aimed at developing genotypes with robust and stable salt tolerance across varying environmental conditions.

### 4.3 Molecular mechanisms, physiological validation, and future directions

To bridge phenotypic screening and mechanistic understanding, this study further validated the results at both molecular and physiological levels using root tissues. qRT-PCR analysis of nine salt response genes (**Figure 7**) revealed that salt-tolerant genotypes exhibited a coordinated transcriptional response characterized by synergistic activation of ion homeostasis and adaptive pathways. Specifically, genes involved in ion transport and homeostasis were more consistently induced in tolerant materials, while adaptative response-related genes also showed a stable upregulation. These patterns indicate that salt-tolerant genotypes can effectively initiate and sustain root-level homeostasis regulation and stress adaptation under salinity. In contrast, salt-sensitive genotypes showed stronger activation of signal and transcriptional regulatory modules (e.g., PP2C-related pathways and transcription factor), but the induction of core ion homeostasis components was inconsistent or insufficient. This indicates that activation of upstream signaling alone is not sufficient for effective salt tolerance (Chawla et al., 2013; Ma et al., 2025b). Rather, the critical determinant lies in the ability to coordinate signaling responses with functional homeostasis mechanisms, ultimately mitigating secondary oxidative stress caused by Na^+^ toxicity and osmotic imbalance (Deinlein et al., 2014; Han et al., 2015; Liu et al., 2021). Consistent with these molecular patterns, physiological analyses (**Figure 8**) further supported mechanistic model. Salt-tolerant materials exhibited enhanced antioxidant capacity (higher SOD, POD, and CAT activities), reduced membrane lipid peroxidation (lower MDA content), and improved osmotic adjustment (higher proline accumulation) under salt stress. In addition, they maintained more effective ion homeostasis by restricting Na^+^ accumulation and promoting K^+^ retention, resulting in a higher K^+^/Na^+^ ratio. Token together, the combined molecular and physiological evidence highlights a coordinated mechanism underlying salt tolerance, involving (i) maintenance of ion homeostasis (Na^+^ exclusion and K^+^ retention), (ii) reduction of oxidative damage, and (iii) stabilization of cellular homeostasis through antioxidant and osmotic regulation. This multi-scale validation not only confirms the reliability of the TOPSIS-based screening framework but also provides a clear mechanistic basis and valuable material resources for future gene discovery, functional analysis, and salt-tolerant breeding.

Although this study established a screening framework combining multiple salt gradients, multiple traits, and multi-scale validation, some limitations remain. First, the analysis focused mainly on the germination and seedling stages, whereas salt tolerance can vary across growth stages (Ahmadizadeh et al., 2016; de Ocampo et al., 2025; Shi et al., 2025). Therefore, seedling performance may not fully reflect adult yield and adaptability (Ahmadizadeh et al., 2016), future studies should validate under pot and field saline alkali conditions. Second, salt stress was stimulated using NaCl, while natural saline-alkali soils involve more complex multi-ion stress and pH effects (Rengasamy, 2016). Future work should incorporate more realistic salt-alkali conditions to improve applicability. Finally, although clear phenotypic differences and stable population structures were identified, the genetic basis remains to be further explored. integrating genotype data through GWAS or QTL mapping could help identify key pathways related to ion homeostasis, antioxidant capacity, and osmotic regulation, providing stronger support for molecular breeding of salt tolerance.

While this study provides a comprehensive multi-trait, multi-gradient evaluation of wheat seedling responses to salt stress, several limitations should be noted. Frist, the screening was restricted to the germination and early seedling stage, which may not fully capture salt tolerance at later developmental stages, including reproductive growth and grain yield. Second, the stress treatment employed only NaCl, where soil salinity in natural environment often involves a mixture of salt, including Na^+^, K^+^, Ca^2+^, and Mg^2+^ ions. Therefore, extrapolation of these results to field conditions should be done with caution, and future studies incorporating multi-ion and full-life-cycle evaluation would enhances the applicability of these findings.

## 5 Conclusion

This study evaluated 120 wheat varieties under four NaCl gradients (0, 50, 100, and 150 mM) and two independent replicates, focusing on key traits including plant height, root length, germination rate, and salt tolerance index (STI). Salt stress significantly inhibited germination and seedling growth and amplified phenotypic differences among varieties, particularly at medium to high salt levels. ANOVA confirmed that salt concentration, genotype, and their interactions significantly influenced trait variation, highlighting both genetic effects and genotype × environment interactions. Correlation analysis further demonstrated that STI is positively associated with major growth traits, supporting its role as a robust integrative indicator for salt tolerance evaluation. Multivariate analyses, including clustering and PCA, revealed stable phenotypic grouping patterns, enabling the identification of representative and extreme materials. TOPSIS-based evaluation provided quantitative ranking of salt tolerance and facilitated the selection of superior germplasm. Molecular and physiological validation further confirmed that salt tolerant materials exhibit coordinated induction of ion homeostasis and adaptive response genes, along with enhanced antioxidant capacity, reduced membrane damage levels, and improved Na^+^/K^+^ balance. Overall, this study establishes a reproducible and scalable integrated multi-gradient phenotypic, statistical analysis, comprehensive ranking, and mechanistic validation. This approach enables efficient identifications of salt-tolerant wheat germplasm and also provides a solid foundation for future genetic and breeding analysis.

## Data Availability

The datasets generated for this study are included in the article and its **Supplementary Material**.
